# Cesium adsorption/desorption behavior of clay minerals considering actual contamination conditions in Fukushima

**DOI:** 10.1038/srep21543

**Published:** 2016-02-12

**Authors:** Hiroki Mukai, Atsushi Hirose, Satoko Motai, Ryosuke Kikuchi, Keitaro Tanoi, Tomoko M. Nakanishi, Tsuyoshi Yaita, Toshihiro Kogure

**Affiliations:** 1 Graduate School of Sciences, The University of Tokyo, Bunkyo, Tokyo 113-0033, Japan; 2 Graduate School of Agricultural and Life Sciences, The University of Tokyo, Bunkyo, Tokyo 113-8657, Japan; 3 Quantum Beam Science Center and Fukushima Environmental Safety Center, Japan Atomic Energy Agency, 1-1-1 Kouto, Sayo-cho, Sayo-gun, Hyogo 679-5148, Japan

## Abstract

Cesium adsorption/desorption experiments for various clay minerals, considering actual contamination conditions in Fukushima, were conducted using the ^137^Cs radioisotope and an autoradiography using imaging plates (IPs). A 50 μl solution containing 0.185 ~ 1.85 Bq of ^137^Cs (10^−11^ ~ 10^−9 ^molL^−1^ of ^137^Cs) was dropped onto a substrate where various mineral particles were arranged. It was found that partially-vermiculitized biotite, which is termed “weathered biotite” (WB) in this study, from Fukushima sorbed ^137^Cs far more than the other clay minerals (fresh biotite, illite, smectite, kaolinite, halloysite, allophane, imogolite) on the same substrate. When WB was absent on the substrate, the amount of ^137^Cs sorbed to the other clay minerals was considerably increased, implying that selective sorption to WB caused depletion of radiocesium in the solution and less sorption to the coexisting minerals. Cs-sorption to WB continued for about one day, whereas that to ferruginous smectite was completed within one hour. The sorbed ^137^Cs in WB was hardly leached with hydrochloric acid at pH 1, particularly in samples with a longer sorption time. The presence/absence of WB sorbing radiocesium is a key factor affecting the dynamics and fate of radiocesium in Fukushima.

Since the accident at Fukushima Dai-ichi Nuclear Power Plant (FDNPP) in March 2011, detailed understanding of the chemical state and dynamics of radiocesium, which is responsible for the high air dose rate in the contaminated area, has been an important issue. Studies conducted before the accident proposed that micaceous minerals are responsible for retaining radiocesium in the soil^1^,^2^. Several groups suggested that illite, a dioctahedral interlayer-deficient mica containing aluminum as the major octahedral cation^3^, is the major sorbent mineral of radiocesium in soil, and they investigated its Cs-sorption properties^4^,^5^,^6^,^7^,^8^,^9^. Some of them^5^,^7^,^9^ suggested that the structure of illite contains several kinds of sorption sites with different affinities and densities. Such results were also reported for actual soils or sediments, from similar Cs-adsorption experiments^10^,^11^,^12^,^13^. In these studies, the highest affinity (but low density) site was suggested to be located at the frayed edges of micaceous minerals, termed “frayed-edge-sites” (FES)^1^,^14^. On the other hand, several works reported that hydroxyl-intercalated vermiculite (HIV) also effectively sorbs and fixes Cs ions^15^,^16^,^17^,^18^. The origin of the high Cs-affinity of HIV is attributed to the wedge-like interlayer transition zone from mica to HIV, which offers sorption sites suitable for Cs and Rb, similar to FES in micas^18^.

On the other hand, Mukai *et al.*^19^ reported that partially-vermiculitized biotite, called “weathered biotite (WB)” in their study, and which originated from the weathering of granite in Fukushima, was a major material in the soil contaminated with radiocesium collected from Fukushima. Komarneni *et al.*^20^ reported the superior sorption property of altered phlogopite such as vermiculite. However, a recent study^21^ which surveyed the Cs-sorption ability of various clay minerals indicated that vermiculite was not superior to the other micaceous clay minerals like illite and smectite. Therefore, a further experiment was expected to discover whether the WB in Fukushima is superior in adsorbing Cs to other clay minerals in Fukushima. First of all, the Cs-adsorption experiments^21^ were performed using a solution with a cesium concentration as low as sub-ppm (~10^−5 ^molL^−1^). However, the Cs-adsorption property of minerals is dependent on the concentration of cesium in the solution and/or the ratio between the amount of the minerals and that of Cs ion in the solution^22^. The actual concentration of radiocesium in the raindrops which caused the radioactive contamination is considered to be very low. For instance, the amount of rainfall in Iitate-village, a seriously contaminated area in Fukushima, in a few weeks after the accident was approx. 10 mm according to the records of the Japan Meteorological Agency^23^. On the other hand, the amount of ^137^Cs per unit area deposited on Iitate-village was ~10^6 ^Bq/m^2^ in the literature^24^. From these values, the concentration in the raindrops should have been in the order of 10 ppt (10^−10 ^molL^−1^). In order to discuss the cesium contamination event in the Fukushima soil, the sorption experiments should be performed using a similar low concentration of Cs. However, this is close to or below the detection limit of the most sensitive analytical instruments. This problem can be solved if radioactive cesium itself is used as the cesium source and the sorption/desorption process is estimated by measuring the radiation from the radioisotope, as was done in previous works^6^,^13^,^22^. Moreover, we evaluated the sorption amounts of ^137^Cs in the minerals by measuring the radiation in the individual mineral particles using quantitative autoradiography with imaging plates (IPs), instead of counting gamma-rays. By using IP autoradiography, we can investigate kinetic aspects of the reaction between a solution and multi-minerals (the details are described later). This approach is considered to reproduce better the reaction between the raindrops and soil that consists of various mineral species actually occurring in Fukushima, than the reaction between a solution and mono-mineral. In the present study, the results of such unique adsorption/desorption experiments for clay minerals possibly present in Fukushima soils are shown. They clearly indicate the importance of WB as the radiocesium sorbent in Fukushima.

## Results and Discussion

### Competitive Cs-adsorption among the minerals

**Figure 1:**
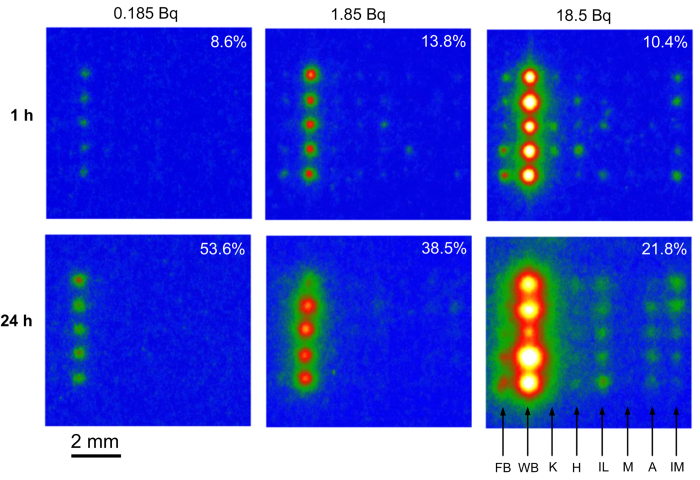
A matrix of the read-out images of IPs covering the substrates with various mineral particles (five particles for each species) sorbed radiocesium from the solutions. The radioactivity input to the solution and reaction time are at the top and left, respectively. The figure at the top-right of each image is the percentage of radioactivity (or ^137^Cs) sorbed to the whole mineral particles, estimated from the IP signal. See Fig. S1 for the arrangement of the mineral particles. The abbreviations at the bottom-right mean FB: fresh biotite, WB: weathered biotite, K: kaolinite, H: halloysite. IL: illite, M: montmorillonite, A: allophane, IM: imogolite.

Eight mineral species, with four or five particles ~50 μm in size for each species, were arranged in the area of a 4 mm by 7 mm square on an acrylic substrate with Kapton double stick tape (Fig. S1). Then, 50 μL solutions containing 3.7, 37 and 370 Bq/mL (0.185, 1.85 and 18.5 Bq in the solutions) of ^137^Cs were dropped to cover all the particles on the substrates. The read-out images of the IPs are presented in Fig. 1. Under any condition with different ^137^Cs concentrations and immersion periods, the amount of ^137^Cs sorbed by WB collected from Fukushima was much higher than the other minerals. In the case of the lower concentrations of ^137^Cs and/or shorter reaction periods, only ^137^Cs was detected at the WB imaging position, showing that WB was the only species sorbing ^137^Cs. At the concentration of 18.5 Bq/50 μL and with a reaction for one day, the sorbed amount of ^137^Cs by WB was about two orders of magnitude higher than the amount of the other clay minerals. These results definitely indicate that WB, which is abundant in weathered granitic soil, sorbs cesium far more than the other clay minerals at a comparable cesium concentration to that for the wet-deposition in Fukushima. This result is consistent with our previous result that WB was frequently found in fine radioactive particles collected from contaminated soil in Fukushima^19^. Our recent investigation^25^ indicated that the radioactive soil particles in the field generally have radioactivity of about the order of 10^−3^ ~ 10^−2 ^Bq, which roughly corresponded to the results in Fig. 1 with a concentration ranging from 0.185 Bq/50 μL to 1.85 Bq/50 μL. It is not certain where the actual sorption sites of cesium are in the structure of WB at such a low concentration level. Considering the extremely low concentration of radiocesium, it is probable that the sorption sites are those with the highest affinity in the mineral. As proposed in previous works^14^,^18^, such sites may be FES or the transition interlayer zone like that in HIV, where the non-hydrated cesium ion with a larger ionic radius than that of potassium resides stably. However, further sophisticated experiments are needed to prove this hypothesis. On the other hand, the Cs-sorption ability of illite, which has been suggested in the previous works^4^,^5^,^6^,^7^,^8^,^9^ to be a dominant Cs-adsorbent in the contaminated soil, was far lower than WB, though it is evident that mineral species alone do not control the sorption property.

**Figure 2:**
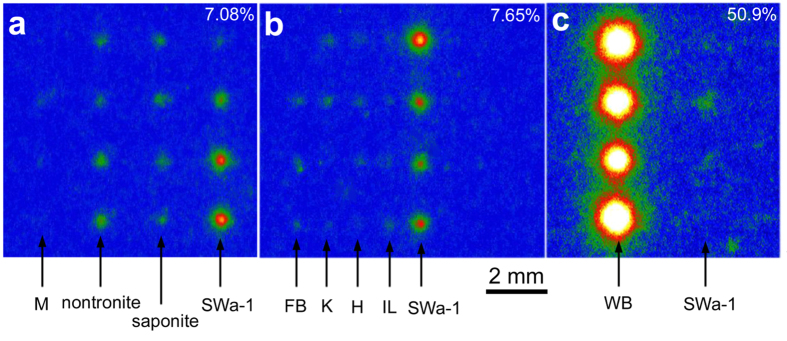
Read-out images of IPs covering the substrates with (**a**) several smectite species, (**b**) ferruginous smectite (SWa-1) and several mineral species other than smectite, (**c**) WB and SWa-1. Four particles were arranged on the substrates for each mineral species. The radioactivity input to the solution was 1.85 Bq and the reaction period was 24 hours. The meanings of the figure at the top-right of each image and abbreviations of the minerals are the same as those in Fig. 1.

In the experiments shown in Fig. 1, we used montmorillonite as the representative of the smectite group. This group includes various kinds of mineral species with different compositions. As is shown in Fig. 1, montmorillonite particles did not appear at all in the IP read-out images, indicating a very weak sorption ability. Hence, we prepared several smectite species and compared their sorption abilities using the same procedure (Fig. 2a). In the experiment, montmorillonite sorbed the least amount of ^137^Cs and ferruginous smectite, SWa-1, sorbed the highest amount of cesium. Saponite and nontronite were at an intermediate level. Such ferruginous smectite was also found in the paddy soil in Iitate-village, Fukushima, as a Cs-sorbent beside WB in our previous work^26^. It was also found that SWa-1 sorbed more ^137^Cs than the other clay minerals except for WB (Fig. 2b). However, the sorption amount to SWa-1 was also negligible when it coexisted with WB on the same substrate (Fig. 2c).

From estimation of the total amount of radiocesium sorbed in the minerals from the IP images, it was apparent that the radiocesium in the solution was not so depleted by the sorption to WB in these experiments (Figs 1 and 2). Hence, it was suspected that the reason for the low Cs amounts sorbed in the other minerals coexisting with WB was low Cs-affinities of these minerals at the low cesium concentration. However, Fig. 2 shows that SWa-1 without WB on the same substrate sorbed far more cesium than that with WB, which implies the depletion of radiocesium by the sorption to WB. An interpretation of this result is that actually most of the cesium was sorbed by WB and the cesium was depleted in the solution, but a considerable amount of sorbed cesium on WB was not tightly sorbed; therefore, it was removed with the solution when the solution was flushed before IP autoradiography.

**Figure 3:**
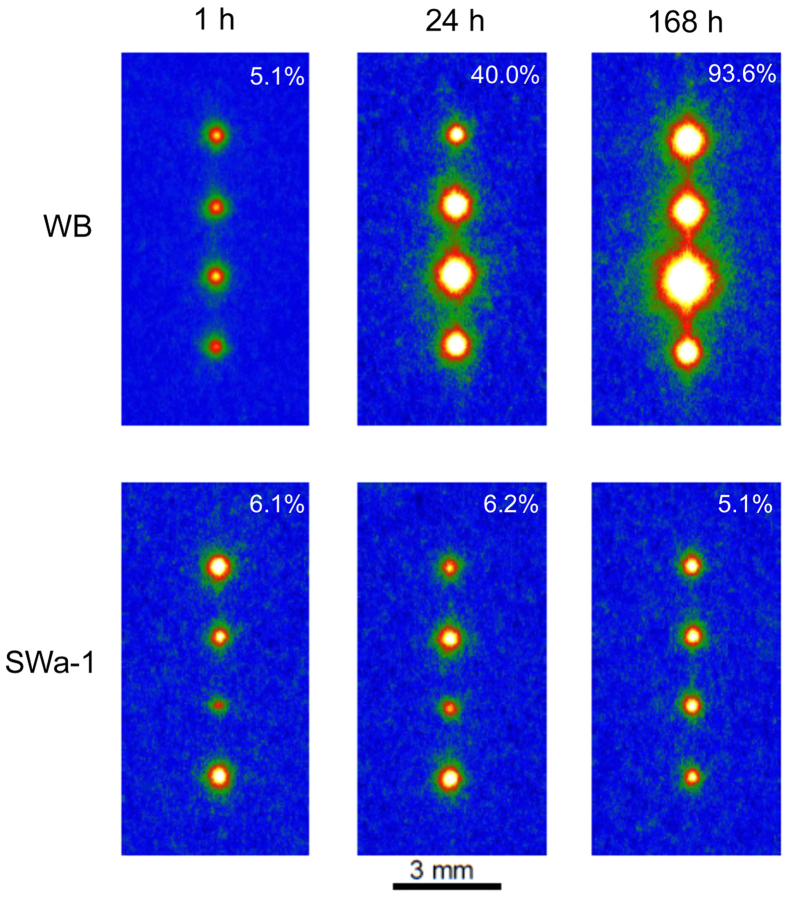
A matrix of the read-out images of IPs covering the substrates with four mineral particles of (top) WB and (bottom) SWa-1, reacted with 1.85 Bq ^137^Cs solution for various periods. Notice that only one mineral species was placed on the substrates and the mineral particles were different for each run.

Besides the amount of the cesium sorbed, the time needed to complete the adsorption was different between WB and SWa-1. Figure 3 shows the IP read-out images of WB and SWa-1 after immersion for one hour, one day and one week, and gives the percentage of ^137^Cs sorbed in the minerals. The amount of cesium sorbed to WB gradually increased with time but the sorption to SWa-1 was almost complete after one hour and further sorption did not occur, suggesting that there were differences of the sorption sites and/or mechanisms between the two minerals. In Fig. 3a, the amount of the sorbed cesium to WB seems to be larger after one week than after one day, showing that the sorption continued for more than one day. However, it should be noted that the mineral particles in each run in Fig. 3 were not the same ones. It is possible that the third WB particle from the top run for one week had an extremely high sorption ability, and the amount of sorption to WB in one day and one week may be comparable.

### Desorption characteristics of WB and SWa-1

**Figure 4:**
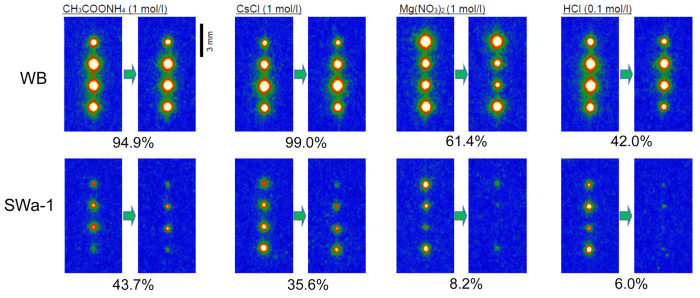
A matrix of the IP read-out images, to show the desorption feature of ^137^Cs from (top) WB and (bottom) SWa-1 using various electrolyte solutions. Initially ^137^Cs was sorbed to the mineral particles from a 50 μL solution with 1.85 Bq for one day. The elution was performed by immersing the substrates in the electrolyte solutions (50 μL) for one day. The figure at the bottom of each image represents the percentage of ^137^Cs remaining in the mineral particles after the immersion.

**Figure 5:**
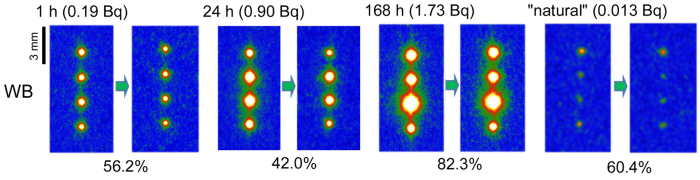
IP read-out images to show the desorption of ^137^Cs by HCl solution (pH 1) from WB particles, which sorbed ^137^ Cs from the solution (18.5 Bq) with different reaction periods, and that from “natural” radioactive WB particles collected in Fukushima. Notice that the images before the elution are the same as those at the top of Fig. 3, except for the “natural” particles. Total radioactivity before the elution is shown at the top, and the percentage of ^137^Cs remaining in the mineral particles after the elution is shown at the bottom.

Desorption experiments were conducted using the Cs-sorbed particles of WB and SWa-1, and various electrolyte solutions for elution. We selected four kinds of electrolyte solutions; 1 mol L^−1^ of ammonium acetate (CH_3_COONH_4_), 1 mol L^−1^ of cesium chloride (CsCl), 1 mol L^−1^ of magnesium nitrate (Mg(NO_3_)_2_) and 0.1 mol L^−1^ of hydrochloric acid (HCl). 1 mol L^−1^ of ammonium acetate is a typical solution to extract ion-exchangeable cations from soil samples^7^,^27^. Cesium chloride was adopted to examine the isotopic exchangeability at the sorption sites. Magnesium nitrate has been reported to efficiently remove cesium sorbed in vermiculite^28^. Finally, 0.1 molL^−1^ (pH 1) of hydrochloric acid was selected to examine whether sorbed cesium in the minerals is desorbed or not with gastric fluid of organism. Desorption was conducted by immersing the mineral particles with sorbed radiocesium in each of these solutions of 50 μL for one day. The results are presented in Fig. 4. Radiocesium in WB was hardy decreased with ammonium acetate and cesium chloride solutions, but was decreased to about half with magnesium nitrate and hydrochloric acid. In contrast, half of the sorbed cesium in SWa-1 was eluted by ammonium acetate and cesium chloride, and almost completely eluted by magnesium nitrate and hydrochloric acid. Finally, the desorption experiments were conducted with WB particles as shown in Fig. 3, to which ^137^Cs was sorbed with reaction times of one hour, one day and one week, and with “natural” radioactive WB particles collected from Fukushima^19^ (Fig. 5). The percentage of cesium desorbed was larger in the particles sorbed for one hour than those sorbed for one week, though the sorbed ^137^Cs amount was much higher in the particles reacted for one week. Although the actual mechanism is not clear, a kind of “aging” effect may exist to increase the affinity of the sorption with time. On the other hand, actual WB particles that sorbed radiocesium in Fukushima released almost half of the radiocesium by the hydrochloric acid solution. Since these particles had been in the field for more than two years, more immobilization of the sorbed radiocesium was expected than that prepared in the laboratory for the one-week treatment, but actually there was no notable difference between the samples from the field and those in the laboratory. The reason is not clear but it might be due to the difference in the weathering stage between the two WB specimens.

In conclusion, the experimental results in the present study clearly indicated that WB can be the major adsorbent of radiocesium if this material is present in the soil, and the dynamics (retention/diffusion) of radiocesium in the soil are strongly dependent on whether WB is present or not. A recent work^29^ reported that the transfer factor of radiocesium from the soil to rice in Fukushima was completely different in soils with and without vermiculite. Our results clearly support the view that such a phenomenon actually occurred in Fukushima.

## Methods

### Mineral Description

WB and its original (fresh biotite: FB) were collected from an outcrop in Ono-City, Fukushima, Japan, where granodiorite of the older type of Abukuma granitic rocks^30^ is exposed. This area is located about 40 km south-west of the FDNPP. The biotite crystals are a few millimeters in width and thickness. The composition of FB was determined to be (K_0.88_Na_0.01_Ca_0.01_)(Fe_1.33_Mg_1.08_Mn_0.02_Al_0.24_Ti_0.16_)(Si_2.83_Al_1.17_)O_10_(OH)_2_, by electron-probe microanalyses. There were various WB crystals with different weathering stages, the variation mainly depending on their location in the outcrop. In this study, we selected the one that appeared the most weathered. Although the chemical composition significantly varied between individual crystals and also inside a crystal (Fig. S2), the averaged composition is roughly expressed as (K_0.16_,Ca_0.07_)(Fe_0.71_Mg_0.64_Al_0.25_Ti_0.22_) (Si_2.67_Al_1.33_)O_10_(OH)_2_, showing a dioctahedral character with the oxidation of iron and leaching of potassium by the hydration of the interlayer, or “vermiculitization”. The XRD patterns of WB and FB are shown in Fig. S3.

For several clay mineral species, clay specimens distributed by the Japanese Clay Science Society (JCSS) were used. They were montmorillonite (JCSS-3101), saponite (JCSS-3501), and illite (JCSS-5101)^31^. The ferruginous smectite and nontronite adopted were SWa-1 and NG-1, respectively, supplied from the source clay minerals of the Clay Mineral Society^32^. The kaolinite specimen originated from Oguni, Yamagata, Japan. X-ray diffraction showed that the specimen was completely mono-mineral with ordered stacking. Halloysite was from Eureka, Nevada, USA^33^. The allophane specimen originated from Kitakami, Iwate, Japan^34^ and the imogolite came from Kurayoshi, Tottori, Japan^35^.

The biotite crystals and consolidated clay minerals were crushed in agate mortar and sieved to 25–53 μm in size. The minerals in a fine powder form were consolidated into a pellet with a hydraulic press, then crushed and sieved to obtain a similar particle size. Typical appearances of these mineral particles are shown in Fig. S4.

### RI solutions

The carrier-free ^137^Cs solution (3.7 MBq mL^−1^ nominal, Eckert & Ziegler Isotope Products, California, USA) was diluted to 370 Bq mL^−1^ (8.4 × 10^−10 ^molL^−1^), 37 Bq mL^−1^ (8.4 × 10^−11 ^molL^−1^), and 3.7 Bq mL^−1^ (8.4 × 10^−12 ^molL^−1^) with reverse osmosis water. Since the ^137^Cs solution is a “reactor produced” isotope product, it probably contains a comparable amount of ^133^Cs with ^137^Cs in the solution.

### Adsorption/desorption experiments

Mineral grains of similar size were selected under a stereomicroscope and arranged on acrylic substrates with Kapton double stick tape, using vacuum tweezers attached to a micro-manipulator (Quick Pro, Micro Support Co., Ltd.) (Fig. S1). Solutions of 50 μL which contained 0.185, 1.85 and 18.5 Bq of ^137^Cs were dropped onto the minerals on the substrate with a micropipette. Then the substrates were enclosed in a styrol box to avoid desiccation, and afterwards, gently flushed with ~10 mL of running water. For the desorption experiments the Cs-sorbed minerals were reacted with four kinds of electrolyte solutions; CH_3_COONH_4_ (1 molL^−1^), CsCl (1 molL^−1^), Mg(NO_3_)_2_ (1 molL^−1^) and HCl (0.1 molL^−1^). The pH values of these solutions were 6.97, 4.26, 5.06 and 1.06, respectively. Each 50 μL solution was dropped onto the substrates and gently flushed after 24 hours.

The adsorbed amounts of ^137^Cs in the minerals were estimated by IP autoradiography. The substrates with minerals were covered with a water-soluble oblate sheet 20 μm in thickness and placed in contact with an IP (BAS-MS, Fuji Film) in the dark for 24 hours. The IP image produced was scanned with an IP reader (FLA-7000, Fuji Film). The read-out images were colored up to be linearly proportional to the IP signal using a Gatan Digital Micrograph 3.10.0. The amount of ^137^Cs in each mineral particle was estimated from the calibration curve between the IP signal integrated over the spot formed by radioactive particles and the radioactivity from the particles measured by a germanium radiation spectrometer (Fig. S5). On the other hand, the total amount of ^137^Cs on a substrate was measured using a germanium radiation spectrometer and compared with the sum of the amount of ^137^Cs for individual particles on the same substrate estimated from the IP signals. The discrepancy between the two measurements was 13%, which indicates the accuracy of the estimation of the amount of ^137^Cs using IP.

## Additional Information

**How to cite this article**: Mukai, H. *et al.* Cesium adsorption/desorption behavior of clay minerals considering actual contamination conditions in Fukushima. *Sci. Rep.* **6**, 21543; doi: 10.1038/srep21543 (2016).

## Supplementary Material

Supplementary Information
