# Supplementary material for: Cesium adsorption/desorption behavior of clay minerals considering actual contamination conditions in Fukushima
Source: Sci Rep. 2016 Feb 12;6:21543. doi: 10.1038/srep21543 (PMC4751473; doi:10.1038/srep21543)
Supplement: Supplementary Information [file srep21543-s1.pdf]

Supplimentary Information for

# Cesium adsorption/desorption behavior of clay minerals considering actual contamination conditions in Fukushima

Hiroki Mukai[1], Atsushi Hirose[2], Satoko Motai[1], Ryosuke Kikuchi[1], Keitaro Tanoi[2], Tomoko M. Nakanishi[2], Tsuyoshi Yaita[3] and Toshihiro Kogure[1,*]

[1]Graduate School of Sciences, The University of Tokyo, Bunkyo, Tokyo 113-0033, Japan

[2]Graduate School of Agricultural and Life Sciences, The University of Tokyo, Bunkyo, Tokyo 113-0033, Japan

[3]Quantum Beam Science Center and Fukushima Environmental Safety Center, Japan Atomic Enegy Agency, 1-1-1 Kouto, Sayo-cho, Sayo-gun, Hyogo 679-5148, Japan

*Corresponding author: Toshihiro Kogure, Department of Earth and Planetary Science, Graduate School of Sciences, The University of Tokyo, 7-3-1 Hongo, Bunkyo-ku, Tokyo 113-0033, Japan
Tel: +81-3-5841-4548
Fax: +81-3-5841-4555
kogure@eps.s.u-tokyo.ac.jp

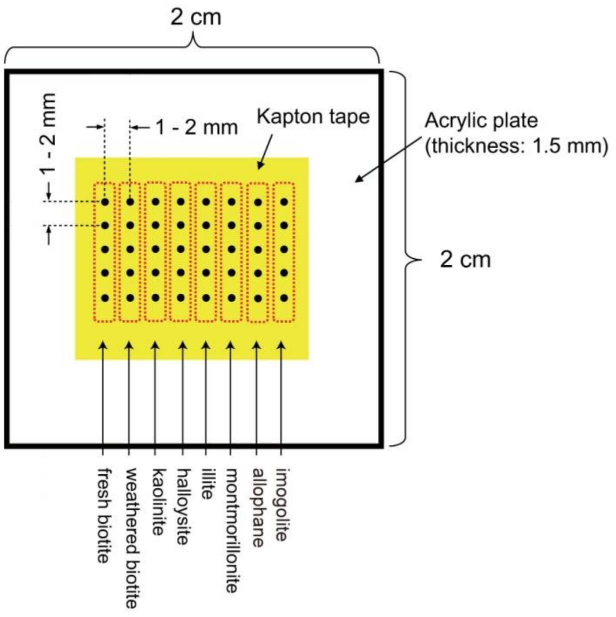


**Figure S1**. A design for the substrate on which fine mineral particles are located with double-stick Kapton tape and a vacuum tweezers on a micromanipulator.

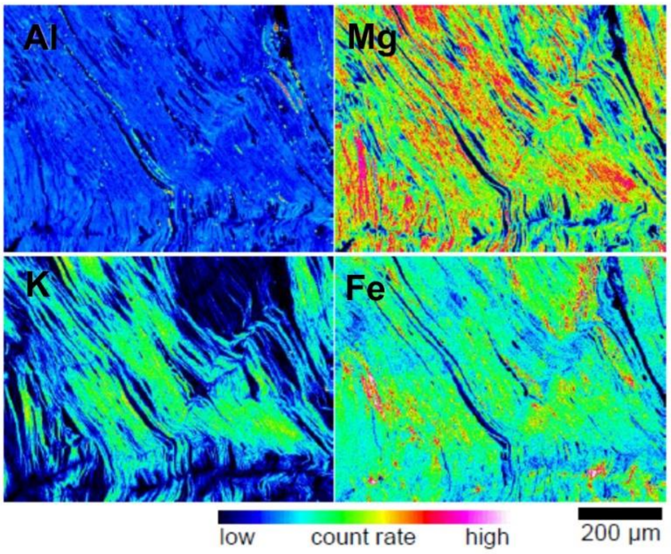


**Figure S2**. Chemical map of Al., Mg, K and Fe from a peterographic thin section of weathered biotite (WB) obtained using an electron-probe microanalyzer.

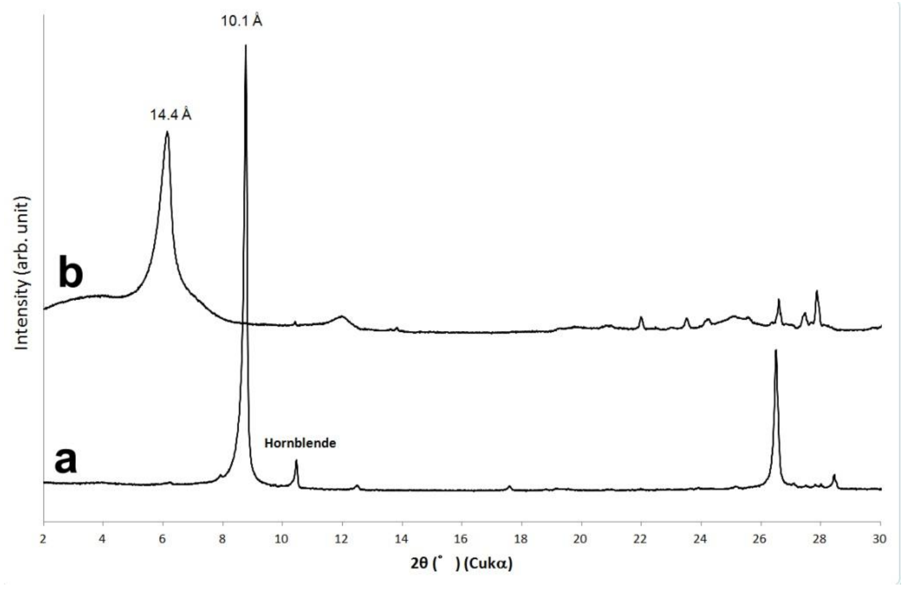


**Figure S3**. X-ray diffraction patterns from (a) fresh biotite (FB) and (b) weathered biotite (WB) used in the experiments.

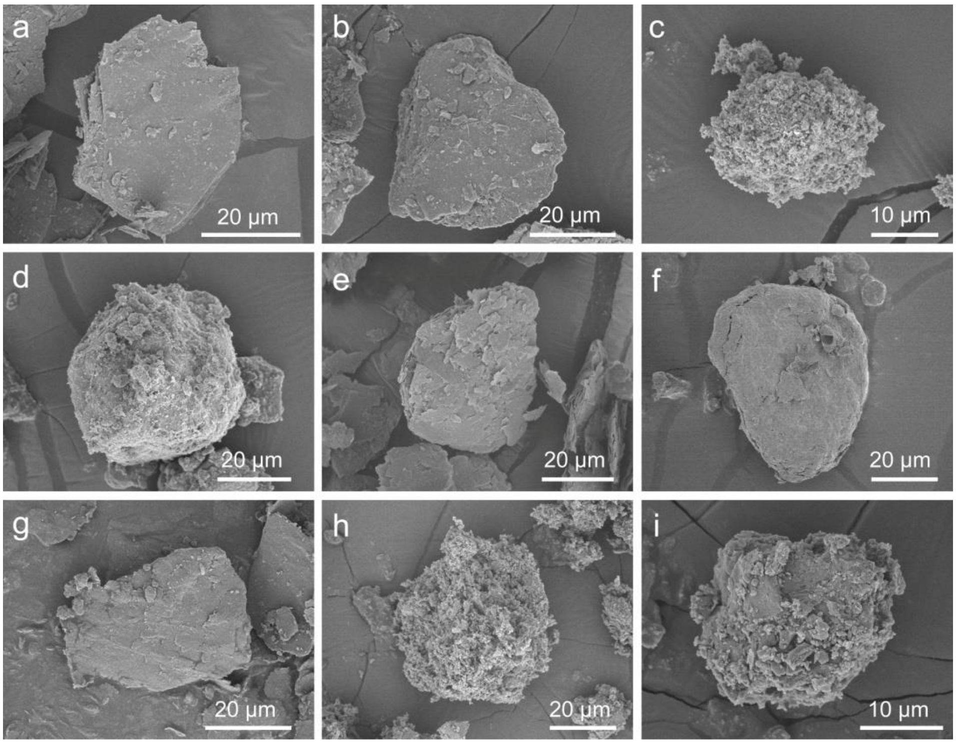


**Figure S4**. Morphologies of the mineral particles; (a) fresh biotite, (b) weathered biotite, (c) kaolinite, (d) halloysite, (e) illite, (f) montmorillonite, (g) Swa-1, (h) allophane, (i) imogolite. The images were taken using a Hitachi S-4500 scanning electron microscope with a field-emission gun operated at 5kV.

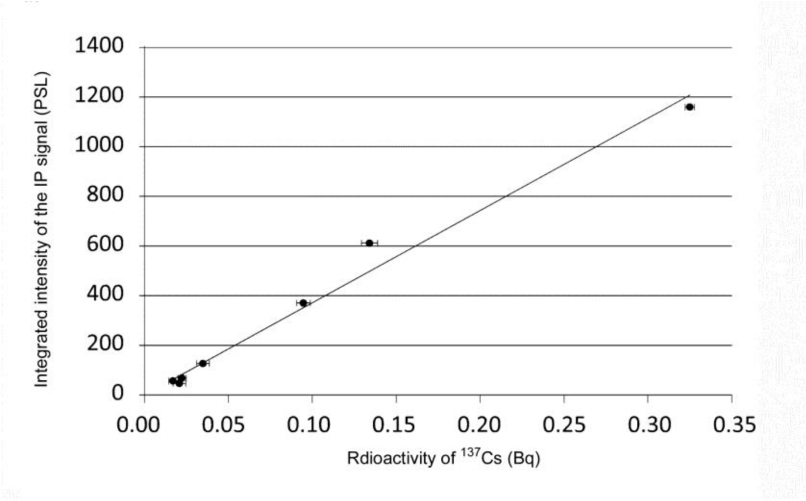


**Figure S5.** Correlation between radioactivity of $^{137}$Cs in fine mineral particles less than 50 μm in size measured using a germanium radiation spectrometer and integrated IP signal intensities inside the spotty images formed by the radiation from the particles on the IP.
